# Supplementary material for: Reservoir controllers design though robot-reservoir timescale alignment
Source: Commun Eng. 2025 Apr 30;4:81. doi: 10.1038/s44172-025-00418-1 (PMC12043989; doi:10.1038/s44172-025-00418-1)
Supplement: Supplementary file 2 — Description of Additional Supplementary Files [file 44172_2025_418_MOESM2_ESM.pdf]

Description of Additional Supplementary Files

File name: Supplementary Movie 1

Description: This video includes the system introduction, the procedure for conducting the timescale alignment test, and two examples for the reservoir computer controlling a cart-pole system.
